# The presence of radioactive heavy minerals in prospecting trenches and concomitant occupational exposure

**DOI:** 10.1371/journal.pone.0249329

**Published:** 2021-03-31

**Authors:** Mohamed Youssef Mohamed Hanfi, Masoud Salah Masoud, M. I. Sayyed, Mayeen Uddin Khandaker, Mohammed Rashed Iqbal Faruque, D. A. Bradley, Mostafa Yuness Abdelfatah Mostafa

**Affiliations:** 1 Ural Federal University, Ekaterinburg, Russia; 2 Nuclear Materials Authority, Cairo, Egypt; 3 Department of Physics, Faculty of Science, Isra University, Amman, Jordan; 4 Department of Nuclear Medicine Research, Institute for Research and Medical Consultations, Imam Abdulrahman Bin Faisal University, Dammam, Saudi Arabia; 5 Centre for Applied Physics and Radiation Technologies, School of Engineering and Technology, Sunway University, Selangor, Malaysia; 6 Space Science Centre (ANGKASA), Universiti Kebangsaan Malaysia, Bangi, Selangor, Malaysia; 7 Department of Physics, University of Surrey, Guildford, Surrey, United Kingdom; 8 Physics Department–Faculty of Science, Minia University, Minia, Egypt; Universidade Federal do Rio Grande - FURG, BRAZIL

## Abstract

Uranium, perhaps the most strategically important component of heavy minerals, finds particular significance in the nuclear industry. In prospecting trenches, the radioactivity of ^238^U and ^232^Th provides a good signature of the presence of heavy minerals. In the work herein, the activity concentrations of several key primordial radionuclides (^238^U, ^232^Th, and ^40^K) were measured in prospecting trenches (each of the latter being of approximately the same geometry and physical situation). All of these are located in the Seila area of the South Eastern desert of Egypt. A recently introduced industry standard, the portable hand-held RS-230 BGO gamma-ray spectrometer (1024 channels) was employed in the study. Based on the measured data, the trenches were classified as either non-regulated (U activity less than 1000 Bq kg^-1^) or regulated (with ^238^U activity more than 1000 Bq kg^-1^). Several radiological hazard parameters were calculated, statistical analysis also being performed to examine correlations between the origins of the radionuclides and their influence on the calculated values. While the radioactivity and hazard parameters exceed United Nations Scientific Committee on the Effects of Atomic Radiation (UNSCEAR) guided limits, the mean annual effective doses of 0.49 and 1.4 mSv y^-1^ in non-regulated and regulated trenches respectively remain well below the International Commission on Radiological Protection (ICRP) recommended 20 mSv/y maximum occupational limit. This investigation reveals that the studied area contains high uranium content, suitable for extraction of U-minerals for use in the nuclear fuel cycle.

## 1. Introduction

The radioactivity of the living environment is contributed to by terrestrial and extra-terrestrial sources. Terrestrial natural radionuclides (^238^U, ^232^Th and their progeny, and ^40^K) exist in all ground formations, forming the main sources of external gamma radiation exposure to humans [1–3]. Extra-terrestrial sources such as cosmogenic radionuclides (^36^Cl, ^32^Si, ^26^Al, ^14^C, ^10^Be, ^7^Be and ^3^H) exist at trace levels, forming a minor part of radiation exposure to humans, albeit at levels varying with altitude and location. About 96% of the total radiation dose to the world population is from these natural sources, while 4% arises from anthropogenic sources [2, 4]. Elevated levels of environmental radiation are contributed to by igneous rocks such as granite, with lower levels typically being associated with sedimentary rocks. Both are used as essential raw materials for building material purposes, landfill etc. The presence of terrestrial radionuclides in raw materials used for constructional intent may pose radiation risks within the living environment [5].

Prospecting for and research on important minerals is being carried out in many parts of the world. Uranium mining in particular is unique in that currently the ores represent the only source of nuclear fuel for nuclear power production [6, 7]. In uranium prospecting, the principal sources of occupational exposure are inhaled radon, thoron, and their respective progenies from the ^238^U and ^232^Th series, along with the associated external irradiation gamma rays [8–12].

Uranium ore deposits in Egypt are found in several areas, including El-Missikate and El- Erediya (in the Central Eastern Desert of Egypt), Gable Gattar (in the North Eastern Desert of Egypt), Abu Rusheid, El Seila and Um Ara (in the South Eastern Desert of Egypt), and Abu Zeneima (South of Sinai) [13, 14]. While numerous studies around the world, including in Egypt, have concerned determination of radiation levels in various trenches or ores as indicators of mineral deposits [15–23], no such investigation has been found for the trenches of the El Seila area of Egypt.

The nomadic El-Bishariya tribe inhabit areas around Seila, practicing a pastoral life-style within this region of the South East Desert. The area is also one with occurrences in the granites of natural uranium, U_Nat_. Over the past several decades, with guidance offered under programes of the Internarional Atomic Energy Agency (IAEA), Egypt has paid great attention to the possibility of using nuclear energy to produce electricity. The responsible national authorities examining justification of such process includes the Nuclear Materials Authority (NMA), undertaking exploration for radioactive raw materials. The NMA has established an exploration project to evaluate and extract radioactive raw materials, primarily from uranium-bearing heavy minerals. Accordingly, many open trenches have been created, workers consequently being exposed, not least from the external gamma radiation from the terresterial radionuclides, either at site or during transportation. Most of the trenches have been located in mountainous or relatively uneven areas, drilling and transportation of the heavy minerals accordingly representing a relatively difficult task. Moreover, weathering, aeration and rock fracturing processes lead to stream sediments, concentrated in various wadis and tributaries, further contributing to radiation exposures, both to surrounding dwellers as well as personnel working in the various prospecting processes.

The present work forms the first such study determining terrestrial radionuclide concentrations and estimating the concomitant radiation exposure inside these particular open prospecting trenches. Several radiological parameters such as the radium equivalent activity Ra_eq_ (Bq kg^-1^), absorbed dose rate in air D (nGy h^-1^), and the annual effective dose AED (mSv y^-1^) have been estimated, seeking evaluation of radiation risk, most particularly to trench workers. Details of the study are presented in the following sections.

## 2. Materials and methods

### 2.1 Environmental setting of the prospecting trenches

**Fig 1:**
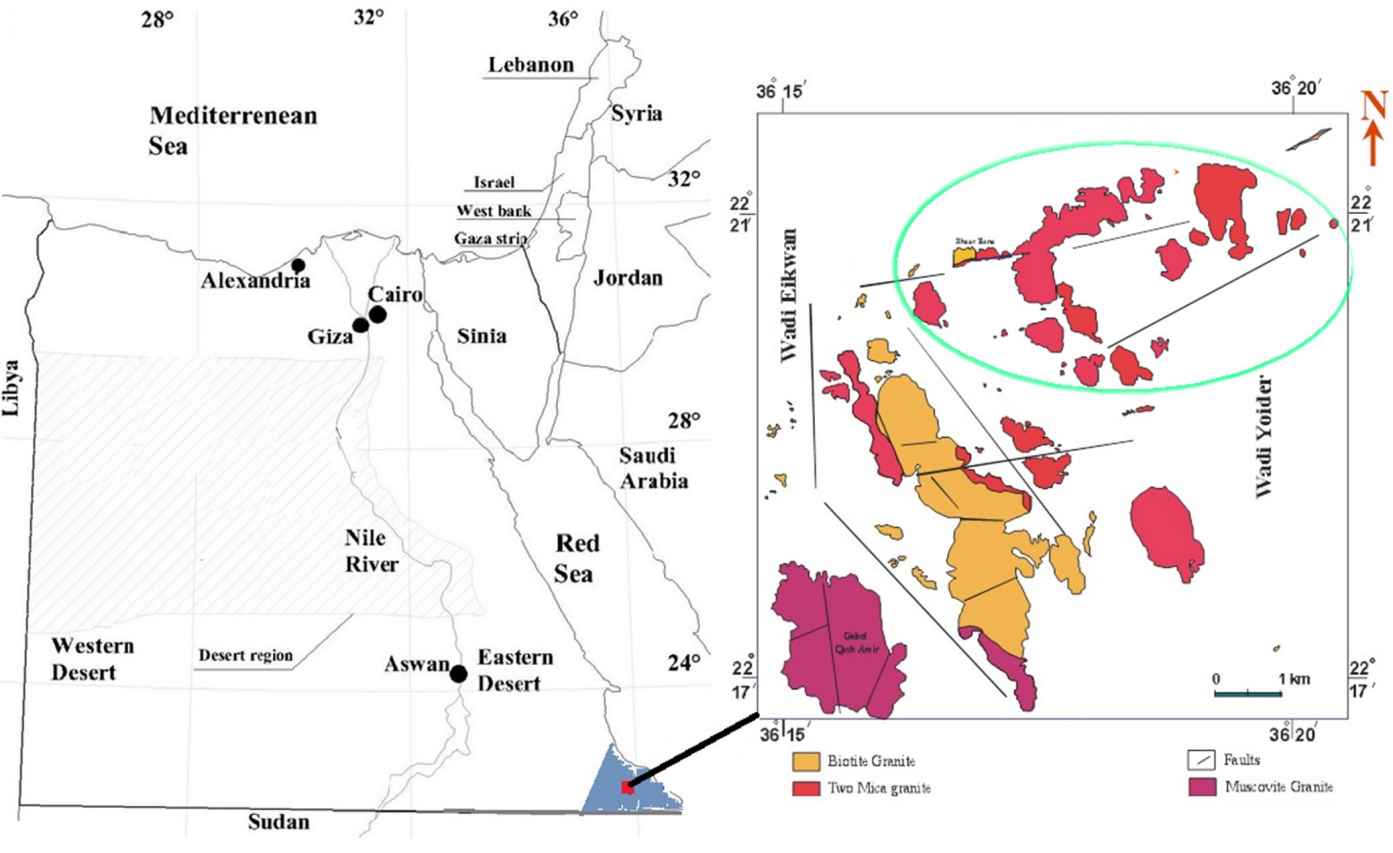
[a] Map of Egypt with the investigated area, [b] the geological map of the Seila area together with the distribution of prospecting trenches [27].

The prospecting trenches in the area of Seila are located between latitudes 22° 13′ 48′′ - 22° 18′ 36′′N and longitudes 36° 10′ 12′′ - 36° 18′ 36′′ E (Fig 1). The granite within these trenches are large highly weathered entities, exposed within low-to-moderately separated hills, being coarse-grained, and pink to pinkish-grey in colour. It is mainly composed of quartz, K-feldspar, plagioclase, biotite, and rare muscovite and is characterized by the presence of iron and manganese oxides filling joints and fractures, indicating Fe and Mn mineralization. The area is further intruded by fine-grained granite, occurring as sheets and dykes trending north-west (NW) to south-east (SE). This granite, dissected by basic dykes, are massive, fine-grained, and range in colour from greyish-green to dark grey, mostly trending east-north-east (ENE) to west-south-west (WSW), usually injected along the extension planes. The southern part of the coarse-grained granite is dissected by a barren quartz vein trending ENE-WSW, extending for more than 600 m, the widest parts ranging from 1 to 10 m [24]. This area is the one in which most of the granite trenches are distributed, perpendicular to the ENE-WSW shear zone. The length of these trenches range from 10 m to 15 m, with width from 5 m to 8 m, and depth of 5–6 m [25, 26].

### 2.2. Sampling method and experimental measurements

A portable, industry-standard RS-230 BGO gamma-ray spectrometer was used to determine the terrestrial radionuclides content in the trenches, popular in uranium exploration. This handheld instrument has been reported to offer convenience and affordability, comparison being made against that of a number of other portable units [28]. Terraplus (2013) showed that a 120 s duration RS-230 BGO measurement provides comparable quality to that performed using the much larger 21 in^3^ NaI portable detector. The spectrometer auto-stabilizes on the naturally occurring radioactivity (K, U, & Th) and does not require any test sources [29].

The present study was carried out as a field study for many regions in Egypt by the Nuclear Materials Authority (NMA). The first and second authors are working in the NMA. This work was done as a part of their MSc. thesis. The area of interest is not restricted or under control, and it is open for measurement by any researchers. The samples were not collected from the prospecting trenches, instead a portable gamma-ray spectroscopy (RS-230) was employed. The RS-230 builds up with a 6.3 in^3^ (103 cm^3^) high density bismuth germinate oxide (BGO) detector. The main characteristics is the recording of readout result rapidly, which facilitates repeated measurements within a short period.

A total of 20 trenches have been studied, obtaining 30 s duration direct readings of ^238^U, ^232^Th, and ^40^K, recorded in ppm, recorded four times for each trench in order to obtain good statistics. The RS-230 BGO spectrometer also provides a measure of the percentage of potassium in the trenches. The radiological measurements were defined by the activity concentration (Bq kg^-1^) instead of content (ppm) [28], both listed in Table 1.

**Table 1:** Conversion of radioelement content to activity concentration (Bq kg^-1^).

Radioelement	Content	Activity concentration (Bq kg^-1^)
^40^K	1% K in rock	313
^238^U	1 ppm U in rock	12.4
^232^Th	1 ppm Th in rock	4.06

Following the recommendation of the IAEA [30], the measured activity concentrations of terrestrial radionuclides in the prospecting trenches have been divided into two categories, non-regulated and regulated. For this, the IAEA has established reference values of 1 Bq/g for radioactive material ^238^U and ^232^Th, and 10 Bq/g for ^40^K; above these values the radioactive material is recommended to be regulated [30].

#### 2.2.1 Assessment of radiation hazard

The key radiological hazard indices were: radium equivalent activity (Bq kg^-1^), absorbed dose rate (nGy h^-1^), and annual effective dose (mSv y^-1^), seeking to evaluate the radiation risk to occupationally exposed workers.

#### 2.2.2 Radium equivalent activity (Ra_eq_)

This index is related to the external gamma-ray exposure and internal dose due to alpha particles, the following formula being used [31]:

$$Ra_{eq}(Bqkg^{-1})=A_{U}+1.43A_{Th}+0.077A_{K}$$ (1)

with *A_U_, A_Th_*, and *A_K_* the activity concentrations of uranium, thorium, and potassium in Bq kg^-1^_._ The weights are based on the estimated dose of 370 Bq kg^-1^ for ^238^U, 259 Bq kg^-1^ for ^232^Th, and 4810 Bq kg^-1^ for ^40^K [32].

#### 2.2.3 Absorbed dose rate

The absorbed dose rate D (nGy h^-1^) is conventionally determined by assessing the gamma-ray exposure at 1 m above the ground surface. This value can be calculated by using the following equation [28, 33]:

$$D(nGy\;h^{-1})=0.427A_{U}+0.662A_{Th}+0.043A_{K}$$ (2)

#### 2.2.4 Annual effective dose

To assess the occupational radiation exposure due to natural radioactivity inside the open prospecting trenches, the annual effective dose (AED) was estimated in mSv y^-1^, the following equation being used [1]:

$$AED(mSv\;y^{-1})=D(nGy\;h^{-1})\times 2000\times 1(Sv\;Gy^{-1})$$ (3)

where the duration of occupational exposure is taken to be 2000 h/y, with a conversion factor of 1 Sv Gy^-1^ for whole-body exposures.

### 2.3. Statistical analysis

Variation in radionuclide activity, in terms of mean and standard deviation was assessed, also data normality, use being made of a one-way analysis of variance (ANOVA-1). SPSS software (SPSS, 2006) was used to test the means by Duncan’s multiple ranges at P < 0.05.

## 3. Results and discussion

**Fig 2:**
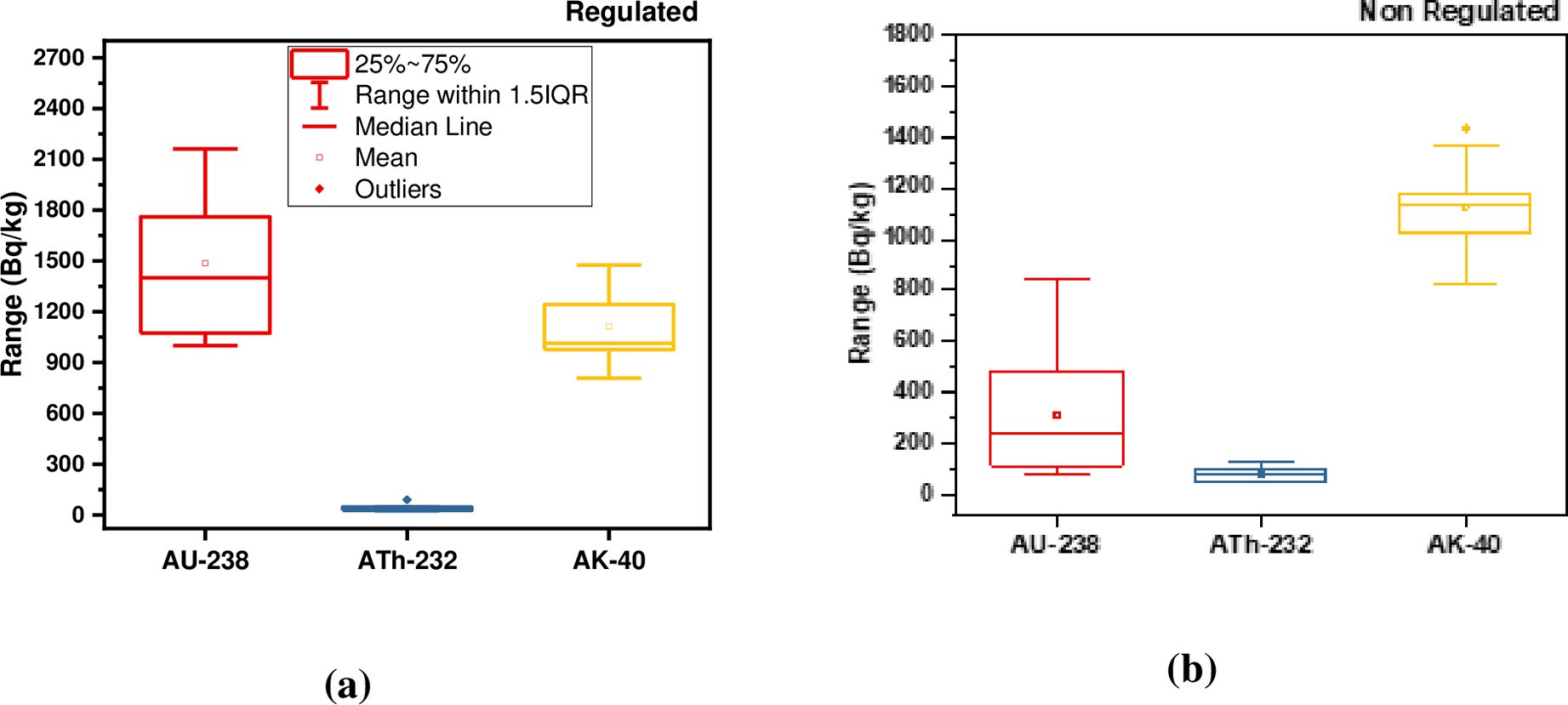
Box and whisker plot of ^238^U, ^232^Th, and ^40^K activity concentrations (Bq kg^-1^) in: (a) the regulated trenches and; (b) non- regulated trenches.

The studied trenches are approximately of the same geometry and physical condition. The average activity concentrations in the two types of trenches (non-regulated and regulated) are shown in Fig 2. For non-regulated trenches, the mean activity concentrations of ^238^U, ^232^Th and ^40^K are 313, 76, 1131 Bq kg^-1^ with ranges (49 to 510 Bq kg^-1^), (42 to 126 Bq kg^-1^), and (805 to 1477 Bq kg^-1^) respectively. For regulated tranches, the average activity concentrations are 1487, 42, 1112 Bq kg^-1^ with ranges (1100 to 2163 Bq kg^-1^), (25 to 92 Bq kg^-1^), and (805 to 1435 Bq kg^-1^) for ^238^U, ^232^Th and ^40^K respectively.

**Fig 3:**
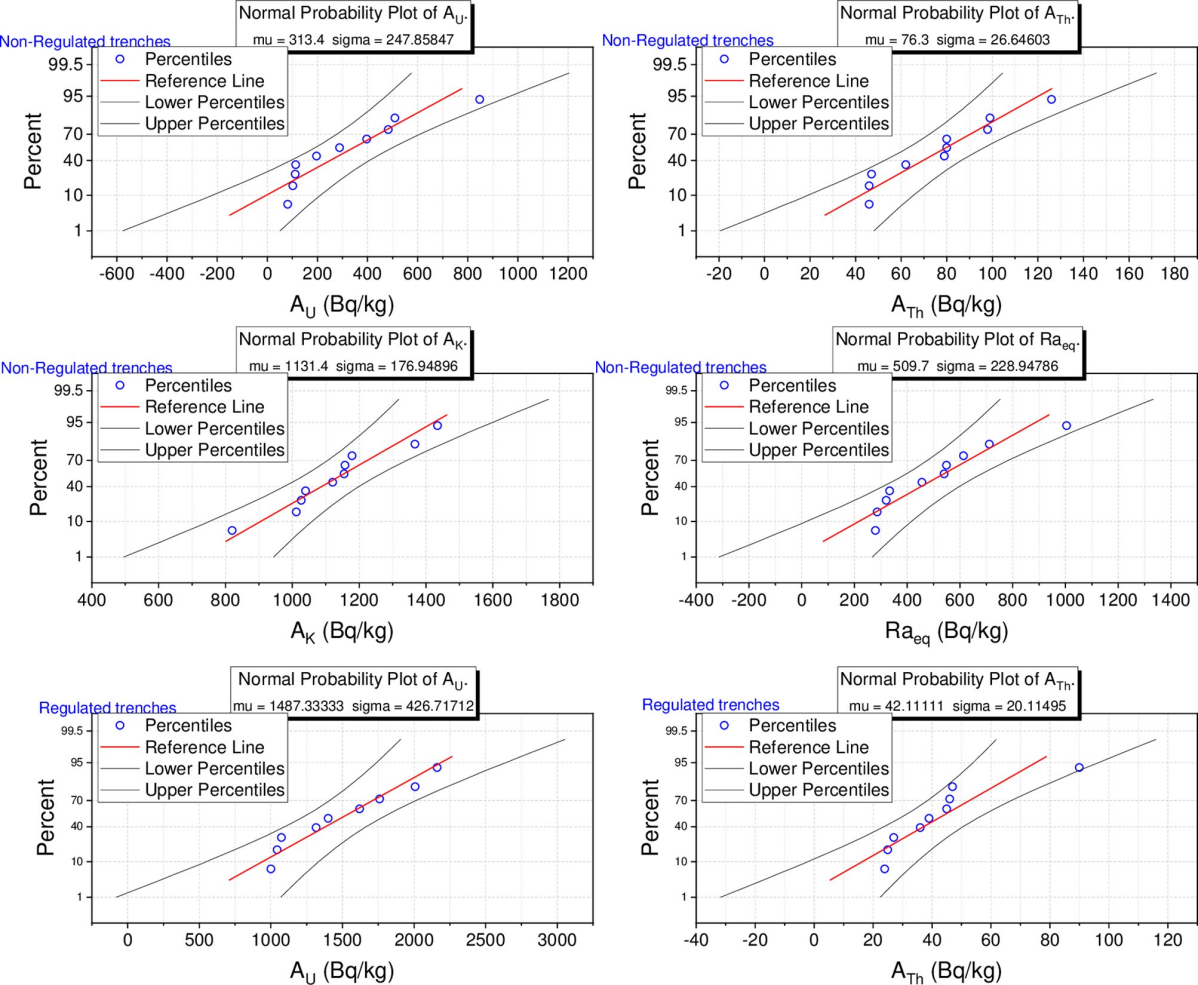
The normal probability of ^238^U, ^232^Th, and ^40^K activity concentrations for the studied non-regulated and regulated trenches.

Overall, the highest activity concentrations of ^238^U, ^232^Th, and ^40^K have been found to be 2163 ± 126, 126 ± 5, and 1476 ± 120 Bq kg^-1^, respectively, while the respective lowest values were observed to be 49 ± 3, 25 ± 2, and 805 ± 15 Bq kg^-1^. These values, other than some small values for ^232^Th, are greater than the UNSCEAR reported reference values of 33, 45, and 412 Bq kg^-1^ for uranium, thorium, and potassium [1]. The various terrestrial radionuclide concentration values in the different prospecting trenches are reflective of geological formation variability, the granite rocks in the studied trenches being considered uriniferous granite in that they contain at least twice the Clarke value [34]. The normal probability of ^238^U, ^232^Th, and ^40^K activity concentrations in the studied regulated and non-regulated prospecting trenches are presented in Fig 3.

**Fig 4:**
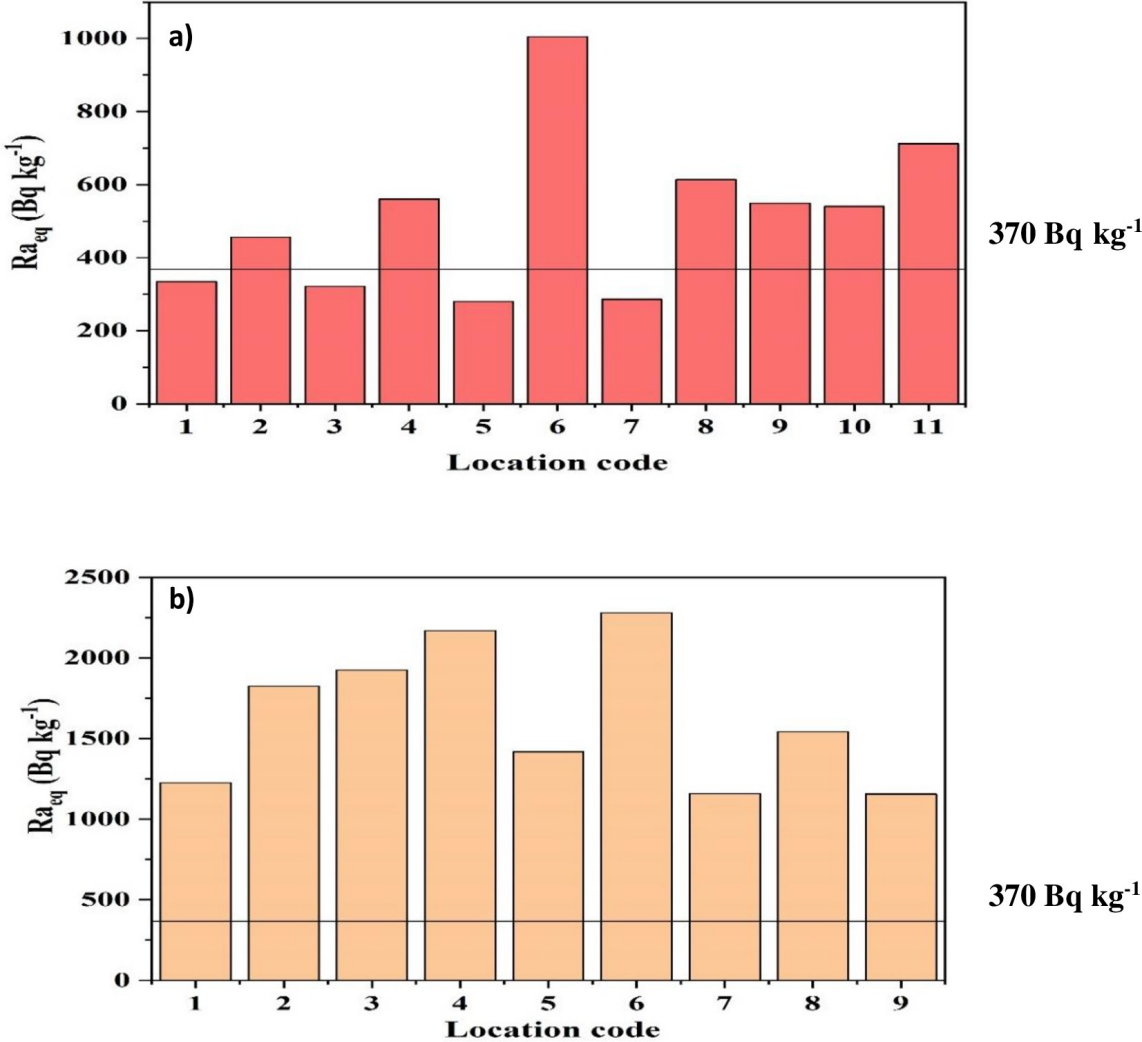
Radium equivalent activity in: (a) non-regulated (a) and; (b) regulated trenches.

The calculated radium equivalent activity (Ra_eq_) values for each studied trenches (both regulated and non-regulated) are presented in Fig 4. The Ra_eq_ values ranging from 282 to 1004 Bq kg^-1^ with a mean of 510 Bq kg^-1^ for the non-regulated prospecting trenches_._ In the regulated prospecting trenches, the range of Ra_eq_ values are 1146–2282 Bq kg^-1^ with a mean of 1634 Bq kg^-1^. The mean values of Ra_eq_, in all of the prospecting trenches these are above the recommended value of 370 Bq kg^-1^ [31], graphically represented in Fig 4. The higher Ra_eq_ belong to granite rocks, rich in potassium together with content of uranium, thorium, and their decay products [35, 36].

In regard to the estimated occupational absorbed dose rates in air (nGy h^-1^), in the non-regulated and regulated prospecting trenches respectively, these range from 131 to 446 nGy h^-1^ with a mean of 241 nGy h^-1^, and from 505 to 982 nGy h^-1^ with a mean of 709 nGy h^-1^. In all of the prospecting trenches these absorbed dose rates are above the recommended value of 59 nGy h^-1^ [1].

**Fig 5:**
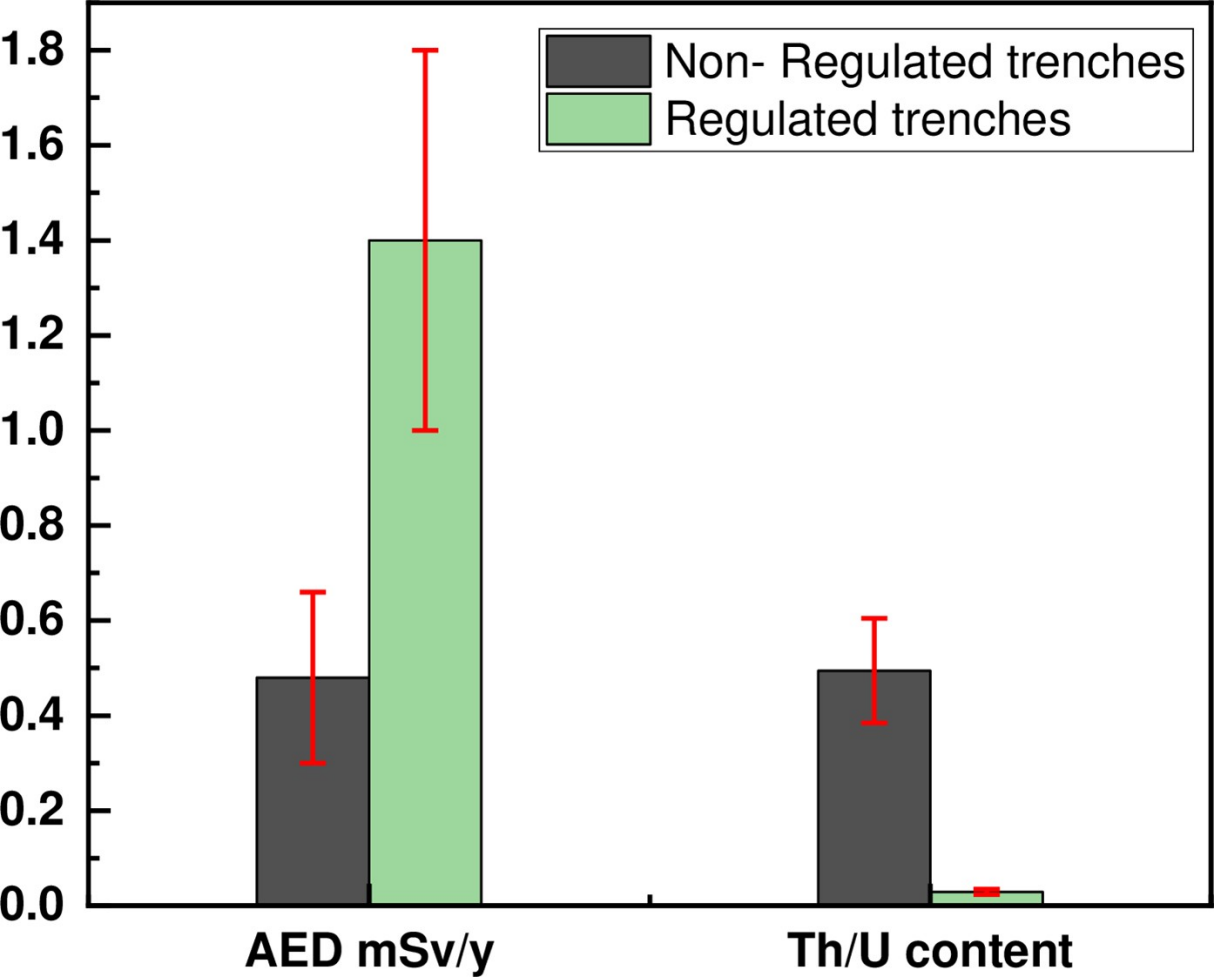
The average AED (in mSv/y) and the Th/U ratio.

The occupational radiation exposure inside the prospecting trenches occurs via two different pathways: external exposure from gamma-rays and internal exposure due to the inhalation of radon and thoron gases and their decay products, attached to atmospheric dust. Present study focuses on the external gamma exposure to workers in the prospecting trenches, with the annual effective dose estimated based on a working period of 2000 h over one year. Fig 5 shows the average annual effective dose (AED) for non-regulated and regulated trenches in the Seila area, with respective values of 0.48 (range 0.27–0.89) mSv y^-1^ and 1.4 (range 1.01–1.97) mSv y^-1^. The occupational AED for regulated trenches exceeds the recommended safe level limit of 1 mSv y^-1^ for members of the public, although it is very much lower than the occupational maximum limit of 20 mSv y^-1^, as mentioned in respect of prospecting trenches [37]. The observed annual effective dose in the trenches showed no significant radiation dose from gamma radiation. Nevertheless, people who live in this area or use the residues as building materials may be exposed to elevated levels of radiation dose.

The ^232^Th/^238^U ratios for non-regulated and regulated trenches in the Seila area are shown in Fig 5, comparison being made with the world average value of 3.94 [38]. The ^232^Th/^238^U ratio for non-regulated trenches ranges between 0.054 and 0.975, with a mean of 0.495. The range for regulated trenches is 0.018 to 0.055, with a mean value of 0.03. These values are below the worldwide average value [39–41], point to the Seila trenches media being igneous zircon [42], containing high uranium-content granitic rocks. Related to variations in stream sediments in terms of geochemical properties, the weather conditions may have affected this ratio. It is worth mentioning that the trenches are located on the high mountains and hills, which surrounded the Seila area. The weather in this area is characterized by rainy in the winter season, therefore the leaching processes could be happened and there is a high possibility that the stream sediments may contributed in various ratios.

The statistical analysis was performed using Pearson correlation for the results in non-regulated and regulated trenches and presented in Tables 2 and 3. Table 2 shows that A_U_ has a strong negative correlation with A_Th_ and A_K_ and a weak negative correlation with the Th/U ratio. A_Th_ has a positive correlation with the Th/U ratio. A_K_ has a negative correlation with A_Th_ and a positive correlation with A_U_. This is due to the relatively high activity of ^238^U and ^40^K.

**Table 2:** Pearson correlation for non-regulated trenches.

	A_U_	A_Th_	A_K_	Ra_eq_	D_air_	AED	Th/U
**A**_**U**_	1	-	-	-	-	-	-
**A**_**Th**_	-0.35	1	-	-	-	-	-
**A**_**K**_	-0.33	-0.36	1	-	-	-	-
**Ra**_**eq**_	0.90	-0.41	0.070	1	-	-	-
**D**_**air**_	0.834	-0.44	0.21	0.98	1	-	-
**AED**	0.84	-0.45	0.21	0.98	0.99	1	-
**Th/U**	-0.87	0.38	0.34	-0.75	-0.68	-0.68	1

**Table 3:** Pearson correlation for regulated trenches.

	A_U_	A_Th_	A_K_	Ra_eq_	D_air_	AED	Th/U
**A**_**U**_	1	-	-	-	-	-	-
**A**_**Th**_	0.29	1	-	-	-	-	-
**A**_**K**_	-0.15	-0.14	1	-	-	-	-
**Ra**_**eq**_	0.99	0.34	-0.12	1	-	-	-
**D**_**air**_	0.99	0.35	-0.11	0.99	1	-	-
**AED**	0.99	0.35	-0.10	0.99	0.99	1	-
**Th/U**	-0.25	0.82	-0.046	-0.20	-0.19	-0.19	1

Table 3 shows that A_K_ has a negative correlation with all other parameters due to the relatively high activity concentration of ^238^U, greater than 1 kBq kg^-1^ and more elevated than the activity of ^40^K itself. A_Th_ has a positive correlation with all other parameters except for A_K_. A_U_ has a strong negative correlation with A_K_ and the Th/U ratio but a weak positive correlation with A_Th_. In both non-regulated and regulated trenches, A_U_ has a strong correlation with the radiological hazard indices (R_aeq_, D_air,_ and AED), approaching unity. This is due to the activity of ^238^U, in all trenches being greater than the recommended limit.

## 5. Conclusions

The Seila region in Egypt is considered as a potential area for exploration of heavy minerals, therefore the prospecting trenches were drilled following some geological and radioactive studies. Present study forms an interest of measuring the concentrations of terrestrial radionuclides in some non-regulated and regulated trenches in this area. The mean activity concentrations of ^238^U, ^232^Th, and ^40^K are found to be 313.4, 76.3 and 1131.4 Bq kg^-1^ respectively in non-regulated trenches, and 1487, 42, and 1112 Bq kg^-1^ respectively in the regulated trenches. In the regulated trenches, with the exception of thorium, the values are greater than that of the worldwide mean data. The estimated radiological indices and concomitant dose show that the workers in the prospecting trenches receive a much lower annual effective dose than the maximum occupational limit of 20 mSv/y, but in regulated trenches the values are above the safe limit of 1 mSv/y for the general population. In order to avoid any unnecessary radiation exposure, measured data suggests the need for control in use of any trench materials/residues for construction of dwellings. The measured concentrations of ^238^U clearly support the viability of heavy mineral (uranium) extraction for practical applications within the nuclear fuel cycle.

## References

[1] UNSCEAR *Sources And Effects Of ionizing Radiation—Exposures of The Public And Workers From Various Sources Of Radiation—UNSCEAR 2008 Report*; 2010; Vol. I; ISBN 9789211422740.

[2] Hanfi M.Y.M. Radiological assessment of gamma and radon dose rates at former uranium mining tunnels in Egypt. *Environmental Earth Sciences* 2019, 78, 113, 10.1007/s12665-019-8089-3

[3] Uosif M.A.M.; Issa S.A.M.; Abd El-Salam L.M. Measurement of natural radioactivity in granites and its quartz-bearing gold at El-Fawakhir area (Central Eastern Desert), Egypt. *Journal of Radiation Research and Applied Sciences* 2015, 8, 393–398, 10.1016/j.jrras.2015.02.005

[4] Khandaker M.U.; Asaduzzaman Kh.; Sulaiman A.F.; Bradley D.A.; Isinkaye M.O. Elevated concentrations of naturally occurring radionuclides in heavy mineral-rich beach sands of Langkawi Island, Malaysia. *Marine Pollution Bulletin* 2018, 127, 654–663. 10.1016/j.marpolbul.2017.12.055 29475708

[5] Hanfi M.Y.; Yarmoshenko I. V.; Seleznev A.A.; Malinovsky G.; Ilgasheva E.; Zhukovsky M. V. Beta radioactivity of urban surface–deposited sediment in three Russian cities. *Environmental Science and Pollution Research* 2020, 27, 40309–40315, 10.1007/s11356-020-10084-9 32656760

[6] Hanfi M.Y.; Yarmoshenko I. V; Seleznev A.A.; Zhukovsky M. V The gross beta activity of surface sediment in different urban landscape areas. *Journal of Radioanalytical and Nuclear Chemistry* 2019, 10.1007/s10967-019-06657-9

[7] Hanfi MY., Yarmoshenko IV, Seleznev A A., Onshchenko A., Z.M. Development of an appropriate method for measuring gross alpha-activity concentration in low mass size fractionated samples of sediment using solid state nuclear track detectors. *Journal of Radioanalytical and Nuclear Chemistry* 2020.

[8] Abdelfatah Mostafa M.Y.; Bader Khalaf H.N.; Zhukovsky M. Radon decay products equilibrium at different aerosol concentrations. *Applied Radiation and Isotopes* 2020, 156, 108981, 10.1016/j.apradiso.2019.108981 31740242

[9] Khalaf H.N.B.; Mostafa M.Y.A.; Zhukovsky M. A combined system for radioactive aerosol size distribution measurements of radon decay products. *Radiation Physics and Chemistry* 2019, 165, 108402, 10.1016/j.radphyschem.2019.108402

[10] Khalaf H.N.B.; Mostafa M.Y.A.; Vasyanovich M.; Zhukovsky M. Comparison of radioactive aerosol size distributions (Activity, number, mass, and surface area). *Applied Radiation and Isotopes* 2019, 145, 95–100, 10.1016/j.apradiso.2018.12.022 30590349

[11] Yuness M.; Mohamed A.; Nazmy H.; Moustafa M.; Abd El-hady M. Indoor activity size distribution of the short-lived radon progeny. *Stochastic Environmental Research and Risk Assessment* 2016, 30, 167–174, 10.1007/s00477-015-1057-x

[12] Yuness M.; Mohamed A.; AbdEl-hady M.; Moustafa M.; Nazmy H. Effect of indoor activity size distribution of222Rn progeny in-depth dose estimation. *Applied Radiation and Isotopes* 2015, 97, 34–39, 10.1016/j.apradiso.2014.12.002 25528018

[13] Bisher A.H. Primary uranium mineralization in paleochannels of the Um Bogma formation at Allouga Southwestern Sinai. *11* *Arab Conference on the Peaceful use of Atomic Energy* 2012.

[14] Hussein H.A.; Abdel-Monem A.A.; Mahdy M.A.; El-Aassy I.E.; Dabbour G.A. On the genesis of surficial uranium occurrences in West Central Sinai, Egypt. *Ore Geology Reviews* 1992, 7, 125–134, 10.1016/0169-1368(92)90008-9

[15] Kolo M.T.; Abdul Aziz S.A.B.; Khandaker M.U.; Asaduzzaman Kh.; Amin Y.M. Evaluation of radiological risks due to natural radioactivity around Lynas Advanced Material Plant environment, Kuantan, Pahang, Malaysia. *Environmental Science & Pollution Research*, 10.1007/s11356-015-4577-5 25925148

[16] Al Amin M.; Khandaker M.U.; Miah M.M.H.; Hossain S. Radioactivity in coral skeletons and marine sediments collected from the St. Martin’s Island of Bangladesh, *Journal of Radioanalytical and Nuclear Chemistry*, 2019, 322, 157–163.

[17] Abedin M.J.; Karim M.R.; Hossain S.; Deb N.; Kamal M.; Miah M.H.A.; et al. Spatial distribution of radionuclides in agricultural soil in the vicinity of a coal-fired brick kiln, *Arabian Journal of Geosciences* 2019, 12, 236.

[18] Khandaker M.U.; Garba N.N.; Rohaizad C.A.H.B.C.; Bradley D.A. Assessment of natural radioactivity levels in stony sand from Black Stone Beach of Kuantan, the Peninsular Malaysia, *Radioprotection* 2019, 54, 211–218.

[19] Öztürk B.C.; Çam N.F.; Yaprak G. Reference levels of natural radioactivity and 137Cs in and around the surface soils of Kestanbol pluton in Ezine region of Çanakkale province, Turkey. *Journal of Environmental Science and Health—Part A Toxic/Hazardous Substances and Environmental Engineering* 2013, 48, 1522–1532, 10.1080/10934529.2013.797242 23802161

[20] Hanfi M.Y.; Mostafa M.Y.A.; Zhukovsky M. V. Heavy metal contamination in urban surface sediments: sources, distribution, contamination control, and remediation. *Environmental Monitoring and Assessment* 2020, 192. 10.1007/s10661-020-8155-z 31823021

[21] Günay O.; Eke C. Determination of terrestrial radiation level and radiological parameters of soil samples from Sariyer-Istanbul in Turkey. *Arabian Journal of Geosciences* 2019, 12, 10.1007/s12517-019-4830-1

[22] Aközcan S. Annual effective dose of naturally occurring radionuclides in soil and sediment. *Toxicological and Environmental Chemistry* 2014, 96, 379–386, 10.1080/02772248.2014.939177

[23] El-Taher A.; Zakaly H.M.H.; Elsaman R. Environmental implications and spatial distribution of natural radionuclides and heavy metals in sediments from four harbours in the Egyptian Red Sea coast. *Applied Radiation and Isotopes* 2018, 131, 13–22, 10.1016/j.apradiso.2017.09.024 29091784

[24] Ibrahim M.E.; Zalata A.A.; Assaf H.S.; Ibrahim I.H.; Rashed M.A. El Sella Shear Zone, Southeastern Desert, Egypt; an Example of Vein-Type Uranium Deposit. *The 9th International Mining*, *Petroleum*, *and Metallurgical Engering Conference*, Cairo University, Egypt, Mining 2005, 41–55.

[25] Abdel-Razek Y.; Masoud M.; Hanfi M.; El-Nagdy M. *Occupational Exposures during the U-Exploration Activities at Seila Area*, *South Eastern Desert*, *Egypt*; 2017; Vol. 50;.

[26] Qureshi A.A.; Tariq S.; Din K.U.; Manzoor S.; Calligaris C.; Waheed A. Evaluation of excessive lifetime cancer risk due to natural radioactivity in the rivers sediments of Northern Pakistan. *Journal of Radiation Research and Applied Sciences* 2014, 7, 438–447, 10.1016/j.jrras.2014.07.008

[27] Shahin H.A.E.R.A. Zr-Y-Nb-REE mineralization associated with microgranite and basic dykes at EL Sela shear zone, South Eastern Desert, Egypt. *Journal of the Korean Physical Society* 2014, 3, 1–12, 10.1186/2193-1801-3-573 25332873 PMC4199964

[28] Abdel-Razek Y.A.; Masoud M.S.; Hanfi M.Y.; El-Nagdy M.S. ScienceDirect Effective radiation doses from natural sources at Seila area South Eastern Desert, Egypt. *Journal of Taibah University for Science* 2016, 10, 271–280, 10.1016/j.jtusci.2015.06.010

[29] Terraplus RS-230 BGO Super-SPEC Available online: www.terraplus.ca,.

[30] International Atomic Energy Agency (IAEA), Vienna, 2018. Naturally Occurring Radioactive Material (Norm VIII), Proceedings of An International Symposium Organized by the Institute of Radiation Protection and Dosimetry, National Nuclear Energy Commission, Brazil in Cooperation With the IAEA and held in Rio de Janeiro, Brazil, 18–21 October 2016.

[31] UNSCEAR Exposures from natural radiation sources (Annex B). *Sources and Effects of Ionizing Radiation* 2000, 84–141, 10.1097/00004032-199907000-00007 10376539

[32] Shuaibu H.K.; Khandaker M.U.; Alrefae T.; Bradley D.A.; Assessment of natural radioactivity and gamma-ray dose in monazite rich black Sand Beach of Penang Island, Malaysia. *Marine Pollution Bulletin* 2017, 119, 423–428. 10.1016/j.marpolbul.2017.03.026 28342594

[33] Kolo M.T.; Khandaker M.U.; Amin Y.M.; Abdullah W.H.B. Quantification and Radiological Risk Estimation Due to the Presence of Natural Radionuclides in Maiganga Coal, Nigeria. *PLoS ONE* 2016, 11(6): e0158100. 10.1371/journal.pone.0158100 27348624 PMC4922655

[34] Maurice Y.T. Uranium in Granites. Geological Society of Canada 1982, *Paper* 81–2.

[35] Abbady A.G.E.; Uosif M.A.M.; El-Taher A. Natural radioactivity and dose assessment for phosphate rocks from Wadi El-Mashash and El-Mahamid Mines, Egypt. *Journal of Environmental Radioactivity* 2005, 84, 65–78, 10.1016/j.jenvrad.2005.04.003 15951069

[36] Abbady A.; El-Arabi A.M.; Abbady A.G.E.; Taha S. Gamma-ray measurements of natural radioactivity in cultivated and reclaimed soil, Upper Egypt. *In* *International Conference on Radioecology and Environmental Radioactivity* 2008, 15–20.

[37] Vennart J. The 1990 recommendations of the international commission on radiological protection. *Journal of Radiological Protection* 1991, 11, 199–203, 10.1088/0952-4746/11/3/006

[38] Baba-ahmed L.; Benamar M.E.A.; Belamri M.; Azbouche A. Natural radioactivity levels in sediments in Algiers Bay using instrumental neutron activation analysis. 2018, 1–10.

[39] Paul D.; White W.M.; Turcotte D.L. Constraints on the 232Th/238Uratio (K) of the continental crust. *Geochemistry*, *Geophysics*, *Geosystems* 2003, 4, 10.1029/2002GC000497

[40] Verdoya M.; Chiozzi P.; Pasquale V. Heat-producing radionuclides in metamorphic rocks of the Briançonnais-Piedmont Zone (Maritime Alps). *Eclogae Geologicae Helvetiae* 2001, 94, 213–219.

[41] Yasmin S.; Barua B.S.; Khandaker M.U.; Kamal M.; Rashid M.A.; Abdul Sani S.F.; et al. The presence of radioactive materials in soil, sand and sediment samples of Potenga sea beach area, Chittagong, Bangladesh: Geological characteristics and environmental implication. *Results in Physics* 2018, 8, 1268–1274, 10.1016/j.rinp.2018.02.013

[42] Heaman L.M.; Bowins R.; Crocket J. The chemical composition of igneous zircon suites: implications for geochemical tracer studies. *Geochimica et Cosmochimica Acta* 1990, 54, 1597–1607, 10.1016/0016-7037(90)90394-Z

